# DNA Electric Charge Oscillations Govern Protein–DNA Recognition

**DOI:** 10.1371/journal.pone.0124444

**Published:** 2015-04-29

**Authors:** Josef Štěpánek, Vladimír Kopecký, Pierre-Yves Turpin, Zhenlin Li, Bernard Alpert, Christian Zentz

**Affiliations:** 1 Laboratoire Jean Perrin, UPMC Université Paris 06, CNRS FRE 3231, Paris, France; 2 ER12, UPMC Université Paris 06, Paris, France; 3 Institute of Physics, Faculty of Mathematics and Physics, Charles University in Prague, Prague, Czech Republic; 4 UR4, UPMC Université Paris 06, Paris, France; Jr, Wake Forest University, UNITED STATES

## Abstract

The transcriptional activity of the serum response factor (SRF) protein is triggered by its binding to a 10-base-pair DNA consensus sequence designated the CArG box, which is the core sequence of the serum response element (SRE). Sequence-specific recognition of the CArG box by a core domain of 100 amino acid residues of SRF (core-SRF) was asserted to depend almost exclusively on the intrinsic SRE conformation and on the degree of protein-induced SRE bending. Nevertheless, this paradigm was invalidated by a temperature-dependent Raman spectroscopy study of 20-mer oligonucleotides involved in bonding interactions with core-SRF that reproduced both wild type and mutated *c-fos* SREs. Indeed, the SRE moieties that are complexed with core-SRF exhibit permanent interconversion dynamics between bent and linear conformers. Thus, sequence-specific recognition of the CArG box by core-SRF cannot be explained only in terms of the three-dimensional structure of the SRE. A particular dynamic pairing process discriminates between the wild type and mutated complexes. Specific oscillations of the phosphate charge network of the SRE govern the recognition between both partners rather than an intrinsic set of conformations of the SRE.

## Introduction

The transcription factor serum response factor (SRF) orchestrates the regulation of genes that encode actin–cytoskeleton and contractile proteins [1] via its binding to a 10-base pair DNA consensus sequence designated the CArG box [2–4]. SRF is a 508-amino acid protein with a core domain of 100 of amino acid residues (core-SRF) that recognizes the CArG box target and recruits ternary transcription factors [5–7]. Core-SRF spans a highly conserved sequence of ~56 amino acids residues, including the MADS box, which defines the MADS family of transcription factors [8]. The functions of the various members of this protein family vary widely, ranging from regulatory factors that control basic cellular processes to those required for the development and determination of cell type [9].

A single, high affinity CArG box is contained within the *c-fos* serum response element (SRE) and involved in the transcriptional regulation of the *c-fos* gene [2, 10]. The core-SRF protein binds as a dimer to the 20-mer oligonucleotide, SRE^fos^, representing the DNA sequence of the *c-fos* SRE [5’-d(GGATGT-C_–5_ C_–4_ A_–3_ T_–2_ A_–1_ T_+1_ T_+2_ A_+3_ G_+4_ G_+5_-ACAT)-3’; CArG box underlined and numbered] [2, 11–13].

Mutations in the *c-fos* CArG box hamper the specific function of SRF [14]. The stoichiometry of the complex with core-SRF is altered [12]: single mutated C_–5_→G (SRE^Gfos^) preferentially forms complexes with the core-SRF at a 1:1 stoichiometry. G→C base mutations at the—5 and—4 positions of the free *c-fos* CArG preserve the B-DNA conformation. The interactions at the mutated positions are modified, generating a shift in the internal dynamics of free oligonucleotides [15]. It remains unknown how these simple changes in DNA sequences may alter protein–DNA recognition.

It has been inferred from biochemical [16, 17] and crystallographic [11, 18] studies that sequence-specific DNA recognition by core-SRF depends on intrinsic and inducible conformational properties of the SRE, i.e., mainly on the large and stable bending of the SRE that is induced by core-SRF fixation. The degree of SRE bending induced by the protein has been considered a major determinant in the recognition by core-SRF and to play a key role in the architecture of higher-order transcription complexes. If this is true, C→G base mutations at positions—5 and—4 of the CArG box of the SRE, in a complex with core-SRF, should exhibit different stable degrees of SRE bending. Raman data of 20-mer SREs embodying *c-fos* CArG box do not bring out the expected signature of various stable bending formations of the oligonucleotide moieties upon forming a complex with core-SRF, although the data reveal conformational heterogeneities in the core-SRF–SRE^fos^ complex, indicating conformer interconversion dynamics of SRE^fos^ ensconced in the core-SRF moiety [19]. Thus, the role of SRE dynamics in the assembly of protein–DNA partners becomes a central question. Using temperature-dependent Raman spectroscopy, we studied the effects induced by the bent–linear interconversion processes of wild type and mutated 20-mer SREs on core-SRF recognition.

## Materials and Methods

### Protein and Oligonucleotides

The present study utilizes the SRF fragments (residues 124–245) expressed and purified, as described previously [12]. The 20-mer oligonucleotide, SRE^fos^, and the two mutants at the 5'-end of the CArG box, SRE^Gfos^ GCATATTAGG and SRE^GGfos^ GGATATTAGG (mutations are underlined), were purchased from Masaryk University (Brno, Czech Republic). The duplexes were prepared as described elsewhere [15, 19]. Core-SRF–SRE complexes were prepared by mixing 10 mL of 5×10^–6^ M of core-SRF with a concentrated solution of the oligonucleotideto yield a concentration of 2.5×10^–6^ M SRE in a 10 mM Tris-HCl buffer (pH 8.5) with 0.1 M NaCl, 1 mM dithiothreitol, and 1 mM EDTA. This solution was concentrated using an Amicon Ultra (Millipore, USA) centrifuge filter with a 10-kDa cut-off until an order of 10^–4^ M was reached for the concentration of the complexes.

### Raman Spectroscopy

Raman spectroscopy was conducted, as previously described [19]. Samples placed in a temperature-stabilized microcell with a 12 μL internal volume were excited using a 488.0 nm line of Ar^+^-laser, and Raman spectra were recorded using a T64000 CCD Raman spectrometer (Jobin-Yvon, France). The effective spectral resolution was ~4 cm^–1^. A neon glow-lamp spectrum was recorded after each analyzed sample, and the Raman shift values were corrected using an automatic recalibration procedure. Spectral contributions of water, Tris (represented by four spectral profiles obtained from Tris spectra measurements at various pH values and concentrations) urea, glass, and the 6^th^ degree polynomial background were subtracted using a least-squares fit. For the core-SRF–SRE complexes, the fit was applied to a difference spectrum between the complex and its free components. Then, the background-corrected spectrum of the complex was determined [19]. The concentrations of core-SRF, the SRE and the complexes were determined as previously described [19]. The spectra were averaged assuming a linear dependence on temperature in the narrow 10-°C temperature range from 5°C to 15°C.

### Stoichiometric Conditions of the Complexes

The equilibrium conditions were determined via titration measurements of the fluorescence anisotropy of the fluorescein-labeled SREs using the core-SRF as a titrant [12]. For the wild type oligonucleotide, the sequential binding of two core-SRF proteins was found with the determined average *K*_d_ value of 6×10^–8^ M. The *K*_d_ values of the core-SRF–SRE^Gfos^ and the core-SRF–SRE^GGfos^ complexes were on the order of 10^–7^ M [12]. Because the concentrations of the complexes reached an order of 10^–4^ M in our experimental conditions, which is more than 1000-fold greater than the *K*_d_ value, the core-SRF–SRE^fos^ complexes measured are under stoichiometric conditions and the SREs non-complexed to core-SRF must be present only in trace amounts due to a possible inaccuracy in the prepared core-SRF to SRE concentration ratio. (The concentrations of the SREs and the core-SRF were determined as previously described [19].) In addition, the single mutated SRE^Gfos^, was previously identified [12] to form 70% 1:1 complexes and only 30% 2:1 complexes at the concentrations used for the fluorescence measurements in our experiments. Therefore a concentration increase of three orders and a 2:1 concentration ratio leads to full formation of 2:1 complexes.

**Fig 1:**
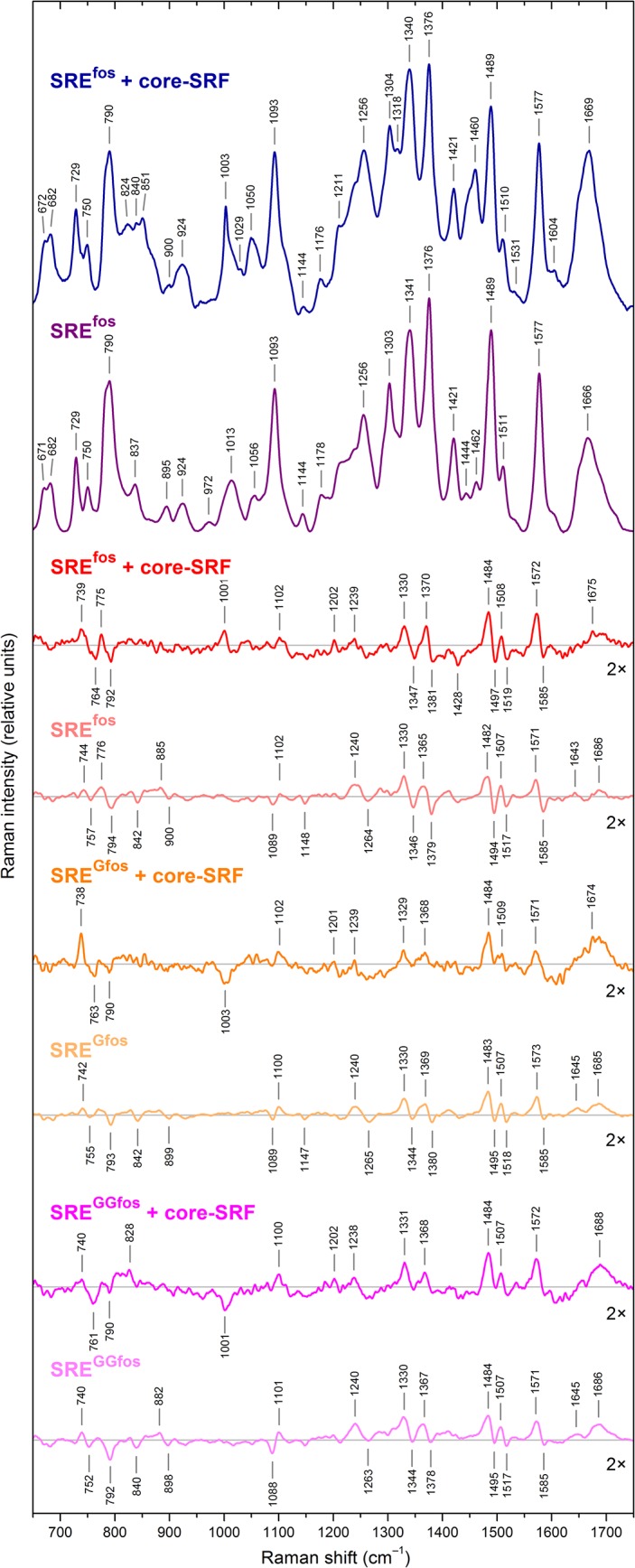
Temperature effect on the Raman spectra of core-SRF–SRE complexes and of free SREs in the premelting domain. The two upper spectra represent the Raman spectra of the core-SRF–SRE^fos^ complex and the free SRE^fos^ at 15°C, respectively. The other spectra are appropriate spectral differences of the Raman spectrum at 15°C minus the spectrum at 5°C. The temperature difference spectrum of each free SRE is below the temperature difference spectrum of its complex with core-SRF.

A possible significant presence of unbound SRE in the Raman spectra was further excluded within the procedure of the spectra treatment (for details, see the previous paragraph). The differential spectra (Fig 1) clearly show that the phosphate vibrational bands at 840 and 1100 cm^–1^ possess quite different temperature-induced changes for the free SREs than those bound to core-SRF. An analogous analysis of the Raman spectra for the core-SRF–SRE^GGfos^ mixture provides the same results, also indicating full complexation at the high concentration conditions for the doubly mutated SRE.

## Results

Temperature-dependent Raman spectra of free SREs [15] reveal two types of changes: a premelting and melting transitions, which are separated by a boundary between 30 and 40°C. The reversible premelting transition expresses a temperature-dependent SRE polymorphism that originates primarily from the central (A/T) domain of the CArG box. The Raman signature is characteristic of an increase in the linear SRE conformer population when the temperature increases. In the premelting domain Raman spectra, the DNA SRE moieties bound to the core-SRF protein exhibit most of the bands that also form in the premelting Raman signature of free SREs, as shown in the difference Raman spectra (i.e., spectra at 15°C minus spectra at 5°C) in Fig 1. The same spectral evolution allows for the common analysis of the temperature effects on the free SREs and on the three individual SRE moieties involved in complexes with core-SRF.

### Flexibility of SRE Moieties Bound to Core-SRF

In the temperature difference spectra shown in Fig 1 the derivative features with peak/trough pairs at 739(+)/764(–) cm^–1^ and 1330(+)/1347(–) cm^–1^ indicate downshifts in the bands at 750 cm^–1^ (dT) and 1341 cm^–1^ (dA), respectively, and correspond to a switch from C2’-*endo*/*anti* to C3’-*endo*/*anti* sugar pucker conformation. The 775 (+)/792(–) cm^–1^ derivative feature indicates that the doublet at 784, 790 cm^–1^ undergoes both a downshift of the 784 cm^–1^ component and an intensity decrease of the 790 cm^–1^ component, expressing changes in the pyrimidine sugar pucker (toward the C3’-*endo*/*anti* conformation) and in the geometry of the phosphodiester group (C–O–P–O–C) [20–22]. The Raman bands corresponding to deoxynucleosides reflect a larger proportion of the 3’-*endo* conformation of the furanose ring at 15°C compared with a larger proportion of 2’-*endo* conformation at 5°C. Thus, more flexible oligonucleotide moieties prevail at 15°C using 5°C as a reference.

### H-Bonding Network of SRE Moieties Bound to Core-SRF

The peak/trough pairs at 1239(+)/1264(–) cm^–1^, 1370(+)/1381(–) cm^–1^, 1484(+)/1497(–) cm^–1^, 1508(+)/1519(–) cm^–1^, and the peak at 1572 cm^–1^ result from downshifts in the Raman bands at 1256 cm^–1^ (corresponding to dT), 1376 cm^–1^ (dT) and 1489, 1511, and 1577 cm^–1^ (all corresponding to dA), respectively. These downshifts are linked to releases of solvent molecules or ions from the (A/T) domain of CArG boxes, and are associated with a weakening of hydrogen bonds inside the protein–DNA complexes, primarily involving adenine and thymine base sites [20, 22].

### Conformational Motions of SRE Moieties Bound to Core-SRF

The high degree of spectral pattern conservation suggests that the same species are present in solution of the three complexed SREs. However, in general, the spectral features of the complexes, from greatest to least specified, follow the order: core-SRF–SRE^fos^ > core-SRF–SRE^GGfos^ > core-SRF–SRE^Gfos^ (Fig 1).

Although the Raman line positions determine the vibrational frequencies of the molecules in the sample, the area and width of the lines are linked to the number and the repartition of these molecular motions. When molecules oscillate between different structures, producing some equilibrium of various conformers, the line widths are highly affected by the respective weights of their populations. Therefore, the Raman spectral shape reflects the distribution of conformers in dynamic interconversion.

Thus, the spectral features express that various constrains between SRE moieties and core-SRF [19] modulate different bent–linear interconversion processes for each of the SRE species. Each complexed oligonucleotide molecule swings continuously, forcing the associated partners to explore different conformational landscapes. The process generates various conformer interconversion populations. This is reflected by the better resolved premelting Raman lines of the SRE^fos^ moiety in the wild type complex compared with the core-SRF, which is characteristic for an interconversion process limiting the width of the conformer distribution. A least-squares analysis of the spectral shapes in the frequency region of 1300 to 1600 cm^–1^ reveals that the temperature difference spectra corresponding to the SRE^GGfos^ and SRE^Gfos^ moieties complexed to core-SRF are on average 4 cm^–1^ and 5–6 cm^–1^, respectively, more broad compared to the temperature difference spectrum of the complexed SRE^fos^. The clear minimum of the smooth dependence on the values of the broadening shows that the internal waving of the SRE^fos^ moiety is less dispersed and its interconversion frequency domain is narrower than those of the mutated SREs. Thus, a particular dynamic pairing occurs in the association between wild type SRE^fos^ and core-SRF. This result highlights the central role played by the SRE conformer interconversion dynamics in the specific protein–DNA recognition.

### Electric Charge Behavior of Phosphate Groups of SRE Moieties Bound to Core-SRF

The three complexed SRE moieties undergo a premelting transition close to, but different from, that of free SREs, which suggests different electric charge ordering.

Indeed, in the temperature difference spectrum of the SRE^GGfos^ moiety bound to core-SRF, the peak at 828 cm^–1^ reflects a downshift in the band at 837 cm^–1^ (phosphodiester marker line), whereas a negative contribution appears at ~842 cm^–1^ in the spectra of free SREs (Fig 1) [15]. Both spectral features reveal a different arrangement of the phosphodiester group torsion associated with the (A/T) domain of CArG box [21]. No contribution of this band appears in the temperature difference spectra of the SRE^fos^ and SRE^Gfos^ moieties, indicating that the phosphodiester torsions of their (A/T) domains are not affected. Consequently, SRE^fos^ and SRE^Gfos^ moieties lead to different spatial and time electric charge organizations with respect to the SRE^GGfos^ moiety and to free SREs.

In the temperature difference spectra of the three complexed SRE moieties (Fig 1), the peak/trough pair observed at 1089(–)/1102(+) cm^–1^ in the temperature difference spectra of the free SREs is mainly replaced by a phosphodioxy (PO_2_^–^) marker band at 1102 cm^–1^ [15, 20, 23]. During the bent–linear interconversion process, a given SRE species bound to core-SRF experiences a set of transient compact states that include the buckling up of phosphodioxy groups and the formation of multiple electric and dipolar configurations [23]. Because the three SRE moieties undergo different bent–linear interconversion processes, each set of multiple electric configurations distinct. Indeed, the variant topologies populate different series of microscopic transient electric charge repartitions, creating different transient electric dipoles specific for each SRE moiety.

Comparisons of the different SRE Raman spectral shapes show that the SRE^fos^ moiety explores a smaller range of conformations and charge distributions than the other two mutated moieties. The temporal fluctuation repartition of the electric network in the wild type complex is less dispersed [19], indicating that the wild type SRE^fos^ association with core-SRF implies a straight frequency domain of the electric dipolar oscillations induced by CArG box motions.

### Core-SRF Moiety Reshaping with Temperature

**Fig 2:**
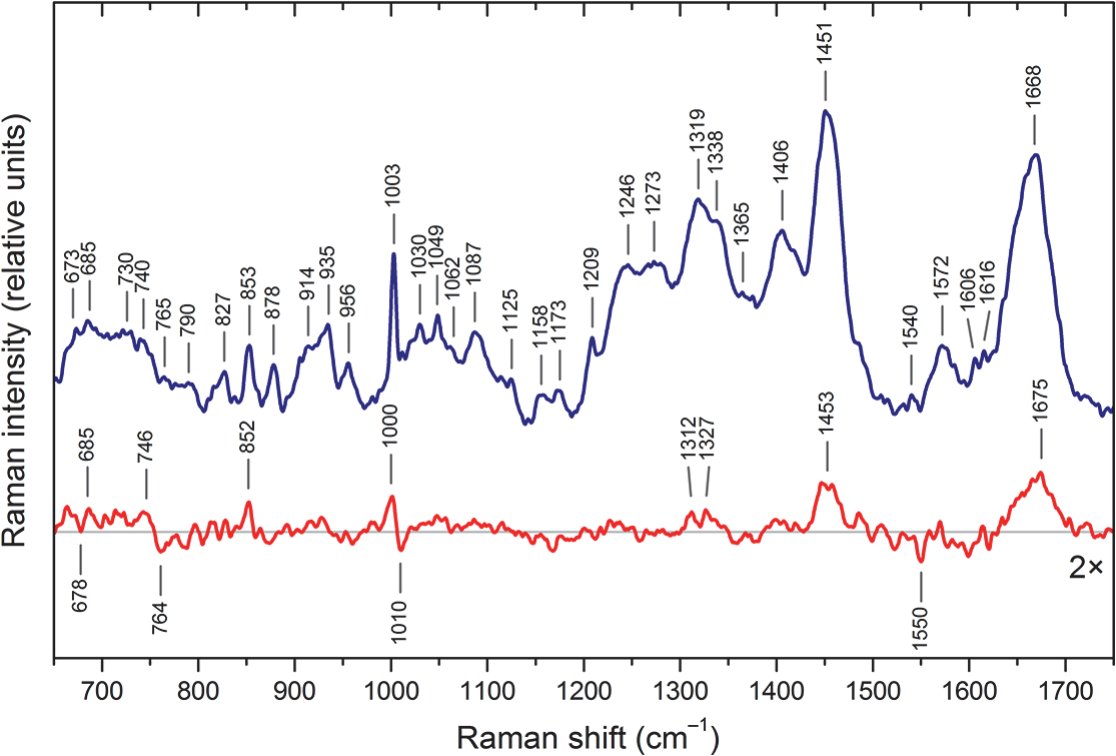
Temperature effect on free core-SRF. The blue curve represents the Raman spectrum of free core-SRF at 15°C. The red curve is the spectral difference between the spectrum of the free core-SRF at 15°C minus the spectrum at 5°C.

Fig 2 shows the temperature dependence of free core-SRF. The most important change consists of a wavenumber upshift from 1668 to 1675 cm^–1^ of the amide I band, suggesting a larger proportion of β-sheets when the temperature increases [24]. In addition, the band at 1453 cm^–1^, assignable to deformation modes of aliphatic side chains, exhibits variations in the hydrophobic packing [24, 25]. In the temperature difference spectra of the core-SRF moieties bound to SREs, the hydrophobic packings are stabilized compared to the free protein, as revealed by the lack of the band at 1453 cm^–1^ in Fig 1.

The temperature difference spectrum of the core-SRF moiety bound to SRE^fos^ (see Fig 1) exhibits a positive band at 1001 cm^–1^ due to phenylalanine, whereas when bound to SRE^Gfos^ and to SRE^GGfos^, the spectra display troughs at 1001–1003 cm^-1^ [26]. This indicates that the changes induced by temperature on the dynamics of the wild type SRE moiety induce a specific strain inside the protein core-SRF, especially located at the Phe residues. Thus, SRE dynamic changes owing to C→G base mutations influence the protein moiety through a redistribution of its strains. Protein-stabilized domains are counterbalanced by motional disorders of some other regions [27, 28].

## Discussion

The temperature Raman approach does not support stable bending of the SRE moieties. Core-SRF binding to SRE^fos^ neither selects nor steadies the bent conformers but involves a continuous switching process between bent and linear conformers of the oligonucleotide moiety. Thus, CArG box sequence-specific recognition by core-SRF cannot be considered simply in terms of the structural SRE bending degree. A combination of coupled structural dynamics and charge movements between both partners is involved in the wild type complex assembly.

### The N-Extension of the αI-Helix is a Determinant of Specificity

In the crystallized core-SRF–SRE complexes, the N-extension of the αI-helix provides a recognition element for the (A/T) domain of CArG box, at least through Arg143, which is buried inside the minor groove [11, 18]. In the vicinity, Thr140 and Arg141 do not make sequence-specific contacts but are also important determinants for binding specificity [18]. The N-extension, which contacts the SRE^fos^ moiety, exhibits a high sensitivity to variations in temperature (high B-temperature factor) [18].

The temperature-dependent Raman spectra show that the constraints exerted on the (A/T) domain, in particular the H-bond repartition, change the flexibility of SRE moieties and, hence, the conformer interconversion processes [15]. Thus, the N-extension dynamics, and the charge of Arg143, affect both the oscillation frequencies and the intensities of SRE^fos^ electric dipoles. By influencing the interconversion dynamics and, consequently, the charge fluctuations of the SRE–DNA moiety, the N-extension of the αI-helix modulates the dynamic pairing and the electric oscillations of the wild type complex. Thus, the N-extension of the αI-helix behaves as a determinant for specificity [7, 29]. In this framework, the relation between charge fluctuations and partner flexibility is an efficient parameter for the protein–DNA association. In other DNA regulatory systems, it has been shown that Arg residues penetrate the DNA minor groove and provide a recognition element for (A/T) sequences, similarly to Arg143 of core-SRF [18]. Many families of DNA-binding proteins involve Arg residues in the minor groove of (A/T) sequences in DNA recognition mechanisms [30].

### Specific Recognition between CArG box and Core-SRF

**Fig 3:**
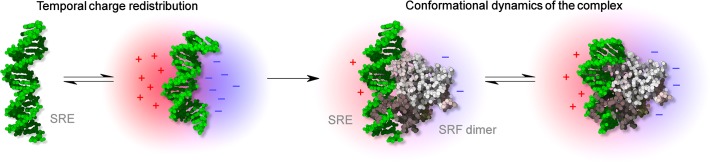
Schematic illustration of the conformational dynamics of the complex with temporal charge redistributions.

Previous studies have inferred that specific binding of core-SRF to the SRE is sequential [12]. In the first step, both partners are attracted by long-range monopolar electrostatic interactions. In the second step, motions of the SRE throughout its conformational landscape generate electric charge oscillations. Transient electric dipoles are thus created in the CArG box of the SRE, which help the charged core-SRF to properly interact with its target in a short range (Fig 3). The crystal of the wild type complex indicates that the molecular two-fold axis of the core-SRF homodimer superimposes with the dyad of the CArG box [11, 18]. Alterations in the DNA dipolar fluctuations involved in C→G mutations at the—5 and—4 positions of the CArG box induce less favorable orientations of protein–DNA charged motifs, thus disturbing the binding efficiency and promoting changes in the core-SRF–SRE^Gfos^ and core-SRF–SRE^GGfos^ complex stoichiometry [12, 15]. Thus, the oriented association between SRE^fos^ and core-SRF requires a convenient spatial and time repartition of transient CArG box dipolar moments, which behave like a fingerprint for the specific recognition between both partners.

It has been suggested that two mechanisms must be combined to achieve the recognition of DNA sequences by the protein, i.e., direct or base readout and indirect or DNA shape readout mechanisms [31]. Owing to close connection between the shape and the electric potential surfaces of DNA, a readout mechanism based on shape supports the DNA charge influence during recognition. Thus, the basic question is no longer how a prerequisite DNA sequence is directly and/or indirectly read by the protein but instead what dynamic scenario involving DNA charge motions leads to stabilization of the core-SRF homodimer on CArG consensus sequences.

### Involvement of SREfos Conformer Interconversion Dynamics in the Stabilization of the Wild Type Complex

The frequency of conformer interconversion dynamics of free SRE is the main parameter in the cohesiveness and the stability of the specific complex [15]. A restricted range of interconversion frequencies allows the charge movements and the structural dynamics inside the wild type complex to be optimized. Conformer interconversion dynamics of the DNA target generates oscillations of an electric field that act as a decisive signal for the association between the protein and DNA. Thus, a specific frequency of the electric field induces a resonance effect between the core-SRF dimer and SRE^fos^. The oscillations of the DNA electric field and the motions of charged amino acids in the protein become intimately linked to one another. In the final stationary motions of the interacting molecules, these oscillations allow a permanent energy exchange between the two partners with very low energy dissipation into their environment. Therefore, the specific protein–DNA complex results from a molecular resonator assembly. This framework permits reveals why static protein conformation alone cannot explain DNA binding activity, as recently demonstrated for the catabolic activator protein (CAP) [32].

### Consequences for the Transcriptional Machinery

Beyond the binary complex, the architecture of the higher-order complexes cannot be considered only from the simplistic point of view of the structural bending degree. A network of coupled structural dynamics and charge movements between partners are likely involved in the extreme spatial flexibility that occurs in ternary complexes between core-SRF–SRE and accessory proteins [33]. The proper frequency of electric field oscillations generated by the dynamics of the DNA target acts as a molecular clutch for the recognition between partners. The present assumptions support recent results indicating that the key point of the transcriptional machinery is an increased durability of the transcription factor complex with the target DNA site with respect to other DNA segments [34]. The binding site occupancy is under the control of its particular electric network oscillations.

## Conclusions

The structural organization alone cannot explain the sequence-specific DNA recognition by the protein. The temperature dependence of the Raman spectra reveals a specific dynamics of the DNA transcription element for both its free form and core-SRF complexes. This dynamics play a key role in the time and spatial repartition of the induced transient dipolar moments of DNA, a cornerstone for the sequence specific protein–DNA recognition and, hence, for the physiological activity of DNA. The conformational arrangement of the protein–DNA complex results from a resonance process that involves more efficient energy exchanges between the protein and DNA than with the environment.
